# NaOH-Only Pretreated Wood Densification: A Simplified Sulfite-Free Route Across Wood Species

**DOI:** 10.3390/polym18030312

**Published:** 2026-01-23

**Authors:** Laura Andze, Vadims Nefjodovs, Juris Zoldners, Ulla Milbreta, Marite Skute, Linda Vecbiskena, Inese Filipova, Martins Andzs

**Affiliations:** 1 Latvian State Institute of Wood Chemistry, Dzerbenes Street 27, LV-1006 Riga, Latvia; vadims.nefjodovs@gmail.com (V.N.); ulla.milbreta@gmail.com (U.M.); inese.filipova@kki.lv (I.F.); martins.andzs@kki.lv (M.A.); 2 Department of Doctoral Studies, Riga Stradins University, Dzirciema Street 16, LV-1007 Riga, Latvia

**Keywords:** wood densification, alkaline pretreatment, sulfur-free processing, mechanical properties (MOE, MOR), hydro-thermo-mechanical processing

## Abstract

The development of high-performance wood-based materials has attracted increasing interest as a means of enhancing the mechanical properties of wood for structural applications. Mechanical densification combined with chemical pretreatment is an effective approach; however, many reported methods rely on complex multi-component chemical systems or severe chemical conditions designed to dissolve lignin or hemicelluloses. In this study, a simplified NaOH-only pretreatment followed by hot-press densification was investigated, targeting selective cell-wall plasticization rather than extensive polymer dissolution. Juniper (*Juniperus communis*), hawthorn (*Crataegus monogyna*), and birch (*Betula pendula*) were used as samples of softwood and hardwood species. Wood specimens were pretreated in 1 M NaOH at 145 °C for 10–30 min and subsequently densified by radial compression. Changes in chemical composition were evaluated by HPLC after acid hydrolysis and FTIR spectroscopy, while microstructural changes were examined using SEM. Physical and mechanical properties were assessed through density measurements and three-point bending tests. The results show that NaOH-only pretreatment induces hemicellulose deacetylation and modification of interpolymer linkages without substantial changes in the main wood polymer contents. Densification resulted in effective lumen collapse and a compact microstructure, leading to a significant increase in density and mechanical properties. Overall, the results demonstrate that efficient wood densification and mechanical enhancement can be achieved by promoting polymer mobility through selective cleavage of interpolymer bonds, using a simplified, single-alkali pretreatment that reduces chemical complexity and material loss while avoiding extensive lignin or hemicellulose dissolution.

## 1. Introduction

The increasing demand for high-performance, lightweight, and renewable structural materials has intensified global interest in lignocellulosic resources as sustainable alternatives to petroleum-derived polymers and energy-intensive metals [1,2]. Wood, a naturally engineered composite of cellulose, hemicellulose, and lignin, offers low density, high specific strength, and excellent environmental compatibility [3,4]. Nevertheless, its broader use in advanced engineering applications is limited by several intrinsic characteristics: moderate density, moisture-driven dimensional instability, and pronounced anisotropy stemming from its hierarchical cellular architecture [5,6]. These constraints reduce the structural reliability of native wood in demanding environments.

Mechanical densification has therefore become a promising strategy for transforming wood into a consolidated lignocellulosic polymeric material with substantially enhanced strength, stiffness, and hardness. Classical thermo-hydro-mechanical densification relies mainly on heat and pressure but often results in incomplete lumen collapse, spring-back, and reduced stability under fluctuating humidity [7,8]. To overcome these limitations, chemical pretreatments are commonly introduced to soften the cell-wall matrix and facilitate irreversible consolidation prior to compression [9].

Although densified wood has been explored for more than a century, the last decade has seen unprecedented progress [10]. A major turning point in the field occurred with the work of Song et al. (2018) [11], who demonstrated that partial delignification via a sulfite-based pretreatment, followed by hot pressing, could produce a structural material with exceptional mechanical performance and dimensional stability. Following this breakthrough, the number of publications on densified wood has expanded in geometrical progression, reflecting increasing scientific and industrial interest in high-performance lignocellulosic materials [10,12].

Recent research covers a wide range of pretreatment chemicals and targeted functionalities. These include sulfite- or sulfite-based delignification to enhance compressive strength and hardness [11,13,14]; deep eutectic solvent pretreatments designed to reduce set-recovery and accelerate processing [15]; and hydrothermal treatments aimed at improving dimensional stability with low chemical input [16,17]. In parallel, densified or delignified wood has become a versatile platform for functional composites, including transparent wood for energy-efficient glazing, wood-based electrodes for energy storage, and high-strength bio-based laminates for lightweight construction [18,19,20]. More recently, highly alkaline and nanostructure-guided approaches have been reported, in which strong alkali concentrations and elevated pressing temperatures are deliberately employed to activate lignin flow and achieve exceptional bonding and strength [21]. Despite this rapid growth, divergent hypotheses persist regarding the optimal levels of lignin removal, hemicellulose retention, and cell-wall plasticization required for maximal densification and mechanical performance.

Furthermore, most high-performance pretreatment routes rely on multi-component alkaline systems (e.g., NaOH combined with sulfite/sulfide) or designer solvents such as deep eutectic solvents. While effective, these systems increase processing complexity, chemical consumption, and waste-handling burdens, limiting their scalability and environmental benefits [16,22,23]. From a mechanistic perspective, such multi-component systems are often designed to induce partial delignification or extensive lignin modification. However, in native wood, cell-wall rigidity and resistance to irreversible deformation are governed not only by lignin content but also by acetyl ester groups in hemicelluloses and by ester- and ether-type linkages within the lignin–carbohydrate complex, which restrict polymer mobility during compression [3,24,25]. Sulfur-containing pretreatments are therefore typically designed for lignin removal [11], whereas hydrothermal treatment in water alone is kinetically limited and proceeds too slowly to enable effective cell-wall plasticization within short processing times [26,27].

Under alkaline conditions, ester saponification and cleavage of labile lignin–carbohydrate linkages occur at lower alkali severities, whereas extensive hemicellulose extraction, lignin dissolution, and cellulose structural transformation are associated with higher alkali concentrations and longer treatment times [28].

At the same time, studies frequently focus on individual species or specific densification regimes, leaving significant gaps in understanding how simplified, low-impact alkaline chemicals affect polymer composition, cell-wall softening, and mechanical behavior across anatomically diverse woods.

To address these gaps, this study explores a streamlined, NaOH-only pretreatment followed by hot-press densification. Three anatomically distinct species—birch (*Betula pendula*), hawthorn (*Crataegus monogyna*), and juniper (*Juniperus communis*)—were selected to evaluate the robustness and generality of this approach. By integrating compositional analysis with FTIR spectroscopy and mechanical evaluation (modulus of elasticity, MOE; modulus of rupture, MOR), this research aims to elucidate the chemical and structural mechanisms underlying densification under simplified alkaline conditions. In contrast to high-severity alkali routes that employ high alkali concentrations and elevated pressing temperatures, milder processing conditions are deliberately employed to reduce process severity and limit extensive thermal modification of wood. Within this framework, the results demonstrate that a minimal-chemical, sulfite-free pretreatment can provide an efficient, scalable route toward high-performance lignocellulosic polymeric materials.

## 2. Materials and Methods

### 2.1. Materials

Solid wood samples of juniper (*Juniperus communis*), hawthorn (*Crataegus monogyna*) and birch (*Betula pendula*) were collected for this study. Juniper samples originated from a forest near the Kegums region, Vidzeme, Latvia. Hawthorn and birch samples were collected in the Tukums region of Kurzeme, Latvia.

Chemicals used for pretreatment included sodium hydroxide (NaOH, >97%, Sigma-Aldrich, Darmstadt, Germany) and deionized water.

### 2.2. Sample Preparation

From each species, defect-free pieces were selected, avoiding knots, cracks or other visible wood defects. All logs were manually debarked, air-dried, and conditioned to uniform moisture content (approx. 8–10%) before pretreatment for one month. From each species, specimens of 90 × 15 × 15 mm (L × T × R) were cut with controlled anatomical orientation. The growth rings and grain direction were arranged so that the radial face remained continuous and unobstructed, thereby allowing subsequent processing to proceed perpendicular to the rings. This ensured a uniform radial plane suitable for later consolidation.

### 2.3. Chemical Treatment

For chemical pretreatment, wood specimens were immersed in an aqueous 1 M NaOH solution.

The NaOH concentration was selected to operate in a moderate alkaline regime, in which sodium hydroxide promotes base-induced deacetylation and modification of labile interpolymer linkages, thereby increasing cell-wall plasticity without extensive dissolution of lignin or hemicelluloses [28]. Comparable alkaline regimes are reported in alkaline pretreatment and sulfur-free pulping-related processes for non-wood lignocellulosic feedstocks, where selective structural modification rather than complete delignification is targeted [29,30].

Each specimen was placed in an individual stainless-steel autoclave containing the solution and allowed to equilibrate for 24 h. The autoclaves were heated in a glycerin bath to 145 °C, after which the cooking process was conducted for 10, 20, or 30 min. The pretreatment temperature was chosen to ensure sufficient reaction kinetics for alkaline modification while limiting excessive thermal degradation of the wood structure; temperatures in the range of approximately 140–150 °C are commonly reported for alkaline pretreatment and non-wood pulping processes [28,31]. Upon completion, the autoclaves were removed and immediately placed in cold water to cool. After cooking, all samples were thoroughly rinsed with deionized water until the effluent reached neutral pH and were subsequently stored in deionized water until further processing. Samples were coded by combining a letter representing wood species—juniper (J), birch (B), or hawthorn (H)—with a number indicating treatment duration (10, 20, or 30 min), yielding composite codes that uniquely identify both material type and processing time.

### 2.4. Densification

Densification was performed according to the previously described protocol by Andze et al. [13] with minor adaptations for the present study. After alkaline pretreatment, specimens were positioned for radial compression and hot-pressed using a single-stage hydraulic press (LAP 40, Gotfried Joos Maschinenfabrik GmbH & Co., Pfalzgrafenweiler, Germany). Pressing was conducted at 5 MPa and 100 °C for 24 h, followed by 12 h of interrupted heating to stabilize the compressed structure and minimize spring-back.

The selected hot-pressing conditions correspond to well-established densification protocols reported in the literature [11]. In the present study, this pressure–temperature regime was combined with NaOH-only pretreatment to achieve effective cell-wall consolidation while deliberately limiting the processing temperature to avoid thermally induced degradation of wood polymers.

Untreated, non-densified samples of each wood species served as controls to assess the effect of NaOH pretreatment and densification. Three specimens were prepared for each condition.

### 2.5. SEM

To analyze the cross-sections of the samples by scanning electron microscopy (SEM), a thin gold coating was applied using a K550X sputter coater (Emitech, South Petherton, UK). The coated samples were then examined with a Vega TC microscope (Tescan, Brno, Czech Republic) using software version 2.9.9.21.

### 2.6. Chemical Characterization

For chemical analysis, untreated and NaOH-treated samples were ground using an M20 mill (IKA-WERKE, Breisgau, Germany).

#### 2.6.1. Extractives

Extractives were quantified by Soxhlet extraction with acetone for 8 h. After extraction, solvents were removed using a PC3001 VARIO rotary vacuum evaporator (Green Vac, Düsseldorf, Germany). Mass measurements were performed with an ES 225SM-DR analytical balance (Precisa, Zurich, Switzerland). Extractives content (*Ex*%*)* was calculated using Equation (1):

(1) $${Ex}_{\%}=\frac{M_{2}-M_{1}}{M_{0}}\cdot 100\%$$

where *M_0_* is the oven-dry mass of the sample, *M_1_* is the mass of the empty oven-dry flask, and *M_2_* is the mass of the oven-dry flask containing the extracted material.

#### 2.6.2. Klason Lignin

Acid-insoluble (Klason) lignin content was determined following the TAPPI T222 om-98 standard procedure [32].

#### 2.6.3. Chemical Composition

Structural carbohydrates and soluble degradation products were analyzed according to the NREL Laboratory Analytical Procedure NREL/TP-510-42618 [33]. Hydrolysates were analyzed using a Shimadzu LC-20A HPLC system (Shimadzu, Tokyo, Japan) equipped with a refractive index detector. Glucose, cellobiose, arabinose, 2-furaldehyde, acetic acid, 5-HMF, levulinic acid, and formic acid were quantified using a Shodex Sugar SH1821 column at 60 °C with 0.008 M H_2_SO_4_ as the mobile phase (0.6 mL·min^−1^). Xylose, arabinose, galactose, and mannose were analyzed using a Shodex Sugar SP0810 column at 80 °C, with deionized water as the mobile phase (0.6 mL·min^−1^). All standards had ≥99.0% purity (Sigma Aldrich, Steinheim, Germany). Prior to injection, samples were neutralized to pH 5–7 with NaHCO_3_ and filtered through 0.45 μm membrane filters.

#### 2.6.4. FTIR

Fourier transform infrared (FTIR) spectroscopy spectra were obtained using a Thermo Fisher Nicolet iS50 spectrometer (Waltham, MA, USA). Spectra were collected in KBr pellets, prepared from 2 mg of finely ground wood and 200 mg of IR-grade KBr (Sigma Aldrich, Darmstadt, Germany). Measurements were recorded from 4000 to 450 cm^−1^, with a resolution of 4 cm^−1^ and 32 scans per sample. All spectra were normalized to their highest absorption peak before comparison.

### 2.7. Physical-Mechanical Properties

#### 2.7.1. Density

The density of each sample was determined after hot pressing and conditioning at 25 °C and 50% relative humidity. Density (ρ) was calculated using Equation (2):

(2) $$\rho =\frac{m}{h\;\cdot \;w\;\cdot \;l}$$

where ρ (kg·m^−3^) is the density, and m (kg), h (m), w (m), and l (m) are the mass, thickness, width, and length of the conditioned samples, respectively.

#### 2.7.2. Three-Point Bending

Mechanical properties were evaluated by determining the modulus of elasticity (MOE) and modulus of rupture (MOR) using a three-point bending test performed on a ZWICK/Z100 universal testing machine (Ulm, Germany), following EN 310 (1993) [34]. The loading span was set to 70 mm, and specimens were tested in the longitudinal direction.

For each sample type, three specimens were tested to determine average MOE and MOR values. The length and width of all specimens remained at their original dimensions (90 mm and 15 mm, respectively). The thickness of untreated control samples was 15 mm, while the thickness of densified samples varied depending on the degree of compression achieved during processing, from 6–6.5 mm for juniper and birch to 8–8.5 mm for hawthorn.

### 2.8. Statistic

Statistical analysis was performed using SPSS 17.0. Mean values (MV) and standard errors (SE) were calculated from three parallel measurements. Differences among groups were evaluated using one-way analysis of variance (ANOVA). Results are presented as MV ± SE, and statistical significance was set at α = 0.05.

## 3. Results

This section presents the results of NaOH-only pretreatment and subsequent densification of birch, hawthorn, and juniper. The effects of pretreatment duration on mass loss and chemical composition are reported first, followed by FTIR and SEM analysis and evaluation of the resulting density and mechanical properties (MOE and MOR).

### 3.1. SEM

**Figure 1:**
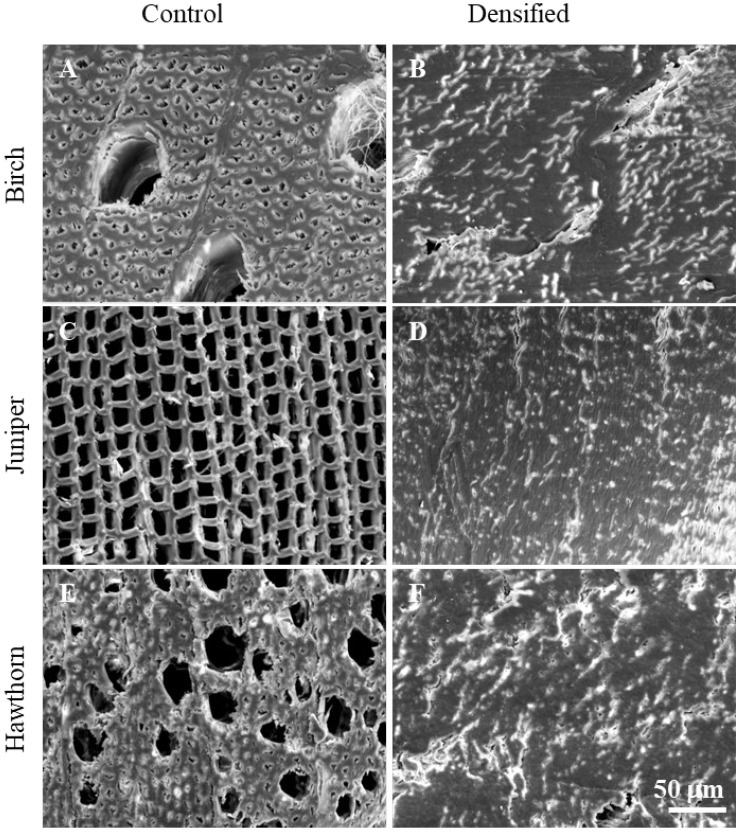
SEM images of untreated birch (**A**), juniper (**C**) and hawthorn (**E**) and densified birch B10 (**B**), juniper J10 (**D**) and hawthorn H10 (**F**) samples at 1000 magnification.

SEM analysis was performed to demonstrate cell-level structural changes in wood (birch—A, B; juniper—C, D; hawthorn—E, F) after chemical pretreatment and densification. A comparison of the microstructures of untreated (A, C, E) and densified (B, D, F) wood samples in the cross-fiber direction at the same magnification is shown in Figure 1.

Figure 1 shows SEM micrographs of untreated wood samples, illustrating typical species-specific anatomical features. The untreated juniper wood (Figure 1C) exhibits a uniform softwood structure composed of earlywood and latewood tracheids arranged in regular radial rows, with relatively consistent lumen sizes and well-defined cell walls [13,35,36]. In contrast, both untreated hardwood samples (Figure 1A,E) have diffuse-porous anatomy [37]. Birch wood (Figure 1A) is characterized by vessels of relatively larger diameter, evenly distributed within a matrix of wood fibers and parenchyma cells [38,39]. Hawthorn wood (Figure 1E) exhibits a finer microstructure, with a higher density of smaller lumens resulting from numerous small vessels and densely packed fiber cells [40,41]. In all untreated samples, open cell lumens and interconnected pore spaces are clearly visible, reflecting the native, non-densified wood structure.

As the microstructural appearance was very similar across all treatment durations, SEM images of samples treated for the shortest NaOH pretreatment time (10 min) are presented to illustrate the representative densified structure. SEM observations of the treated samples indicate that all chemically treated and densified woods exhibit a compact microstructure, in which pores, vessels, and tracheids are nearly completely collapsed and closely packed (Figure 1B,D,F).

### 3.2. Chemical Characterization

The chemical composition of the samples before and after NaOH treatment and densification was characterized using HPLC analysis after acid hydrolysis, Soxhlet extraction for extractive content determination, and the Klason lignin method for quantification of acid-insoluble lignin.

As shown in Table 1, untreated juniper wood contains a higher amount of extractives (~3%), whereas hawthorn (~1%) and birch (~1.5%) exhibit lower extractive contents, which is typical of hardwood species. Accordingly, NaOH treatment led to a pronounced reduction in the extractive content of juniper wood, decreasing from 2.8% after 10 min (J10) to 0.65% after 30 min (J30). In contrast, the extractive content of hawthorn wood remained essentially unchanged after NaOH treatment. For birch wood, NaOH treatment resulted in a moderate decrease in extractives, reaching approximately 1%. As shown in Table 1, the total hemicellulose content of untreated wood was approximately 21% for juniper, 28% for hawthorn, and 32% for birch. After NaOH treatment, the total hemicellulose content of juniper and birch wood decreased by approximately 1%, while a decrease of about 2% was observed for hawthorn. Juniper hemicelluloses are characterized by the presence of both xylan and mannose, with relatively high contents of galactose and arabinose, which is typical of softwood species. In contrast, hawthorn and birch exhibit a predominance of xylose with comparatively lower amounts of other hemicelluloses, consistent with the hemicellulose composition characteristic of hardwoods [42].

Juniper exhibits a high initial lignin content of approximately 31%, which is consistent with values reported in the literature [13,43]. In contrast, untreated birch and hawthorn wood contain about 22–23% acid-insoluble lignin, which is typical for hardwoods [44,45]. After NaOH treatment, even at longer treatment times, the lignin content remained largely unchanged, decreasing by only about 1%.

The most pronounced changes after NaOH treatment are observed in the potential acetic acid content released after acid hydrolysis, as determined by HPLC. The potential acetic acid content decreased from 1.8, 3.6, and 2.2% to 0.1, 1.2, and 0.8% for juniper, hawthorn, and birch, respectively.

### 3.3. FTIR

**Figure 2:**
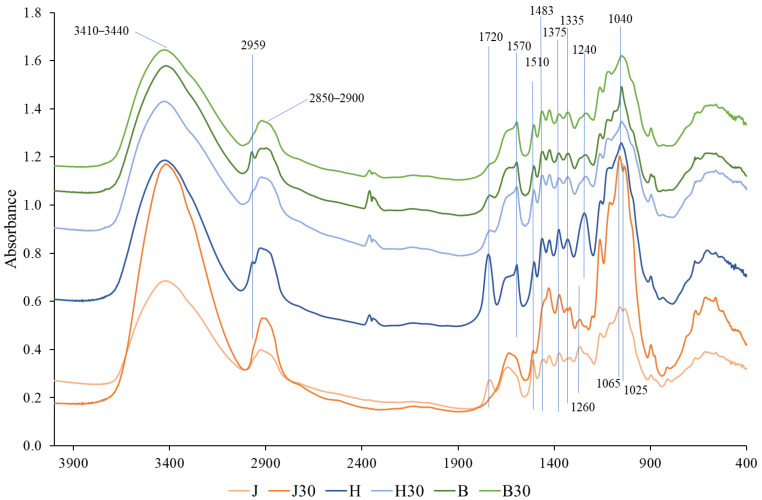
FTIR spectrum of untreated juniper (J), hawthorn (H), and birch (B), and at different times (30 min), NaOH-treated densified wood.

To investigate the effects of wood species and chemical pretreatment on cell wall polymers, FTIR spectra of untreated and treated samples were compared (Figure 2). The FTIR absorption band at 3410–3440 cm^−1^ corresponds to O–H stretching vibrations associated with inter- and intramolecular hydrogen bonding, which are characteristic of cellulose and hemicelluloses. The bands at approximately 2900 and 2850 cm^−1^ correspond to asymmetric and symmetric C–H stretching vibrations of aliphatic methylene (–CH_2_–) groups originating from cellulose, hemicelluloses, lignin, and extractives [46,47]. No significant changes were observed in these spectral regions after NaOH treatment, indicating that no substantial alterations occurred in the cellulose and hemicellulose structures, which is consistent with the HPLC results presented in Table 1. Of particular interest are the absorption bands at ~2959 and ~1720 cm^−1^. The band at ~2959 cm^−1^ is mainly observed in the hardwood samples, whereas the band at ~1720 cm^−1^ is present in all untreated wood samples, including juniper [48,49]. Both bands decrease significantly or even disappear after NaOH treatment.

The absorption band at ~1570 cm^−1^ is characteristic of syringyl-type lignin units, which are typical of hardwood species. In contrast, the band at ~1510 cm^−1^ and 1483 cm^−1^ is mainly associated with aromatic skeletal vibrations of guaiacyl-type lignin [50,51,52]. Softwoods, such as juniper, contain predominantly guaiacyl lignin, whereas hardwoods are characterized by the presence of both syringyl and guaiacyl lignin units, with syringyl lignin being the dominant component [53]. After NaOH treatment, noticeable decreases in the intensities of the ~1510 and ~1483 cm^−1^ bands are observed only in the juniper samples. The absorption bands at ~1375 and ~1335 cm^−1^ are associated with C–H bending vibrations of carbohydrate structures, mainly cellulose and hemicelluloses [47,54]. No significant changes in the intensity of these bands are observed after NaOH treatment, indicating that the carbohydrate backbone remains largely unaffected. This observation is consistent with the HPLC results (Table 1), which indicate only a minor decrease in total carbohydrate content following treatment. The absorption bands at ~1240 and ~1260 cm^−1^ are associated with C–O stretching vibrations in lignin and reflect differences in lignin composition between hardwood and softwood species. Hardwoods show a more pronounced band at ~1240 cm^−1^, related to syringyl-type lignin, whereas juniper exhibits a dominant band at ~1260 cm^−1^ associated with guaiacyl-type lignin [51,55]. No significant changes in these bands are observed after NaOH treatment, indicating that the relative lignin composition remains largely unchanged, consistent with the results in Table 1. In the polysaccharide fingerprint region, hardwood samples show a dominant band at ~1040 cm^−1^, which is characteristic of xylan-rich hemicelluloses [47]. In contrast, juniper exhibits two distinct bands at ~1065 and ~1025 cm^−1^, which are associated with glucomannan-type hemicelluloses [56]. No substantial changes in this region are observed after NaOH treatment, indicating that the main carbohydrate backbone structures are preserved mainly, consistent with the minor changes in sugar composition determined by HPLC (Table 1). Extractives constitute only a minor fraction of the total wood mass and were quantified gravimetrically by Soxhlet extraction. Because of their low abundance, changes in extractive content are not readily discernible in FTIR spectra, as the dominant contributions of the main wood polymers mask their vibrational signals.

### 3.4. Mechanical Properties

**Figure 3:**
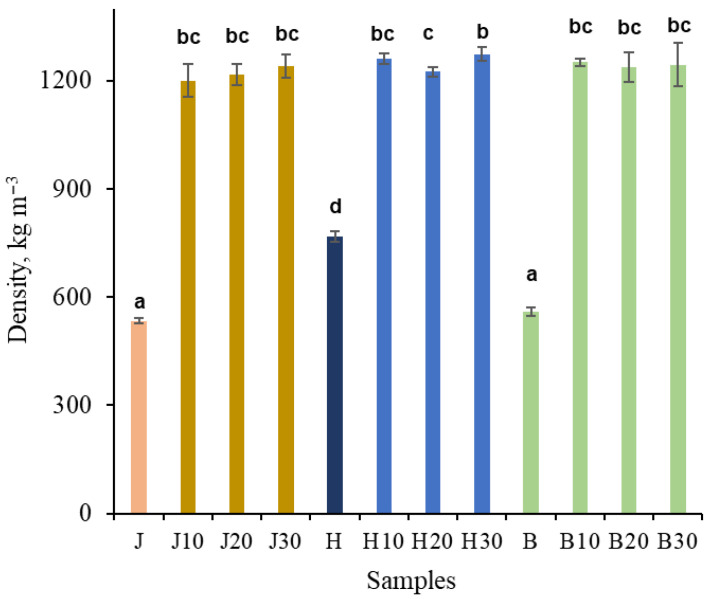
Density of untreated juniper (J, orange), hawthorn (H, blue), and birch (B, green), and at different times (10, 20, 30 min), NaOH-treated densified wood. Bars sharing the same letter showed no significant difference by ANOVA at *p* = 0.05 (*n* = 3).

The initial density of the untreated samples was 560 and 535 kg m^−3^ for birch and juniper, respectively, while hawthorn exhibited a higher initial density of 770 kg m^−3^ (Figure 3). NaOH pretreatment followed by densification resulted in a substantial increase in density for all wood species.

Compared with the untreated state, the density increased approximately 2.5-fold for birch and juniper, whereas hawthorn increased approximately 1.6-fold, which is attributable to its significantly higher initial density. After NaOH pretreatment followed by densification, all samples reached densities of approximately 1250 kg m^−3^, with no statistically significant differences observed between wood species or pretreatment durations. The only exception was the hawthorn H20 and H30 samples, which exhibited a statistically significant difference between them. The achieved density of the densified wood is close to the theoretical maximum wood density of ~1500 kg m^−3^, which corresponds to the density of the wood cell wall material [57].

**Figure 4:**
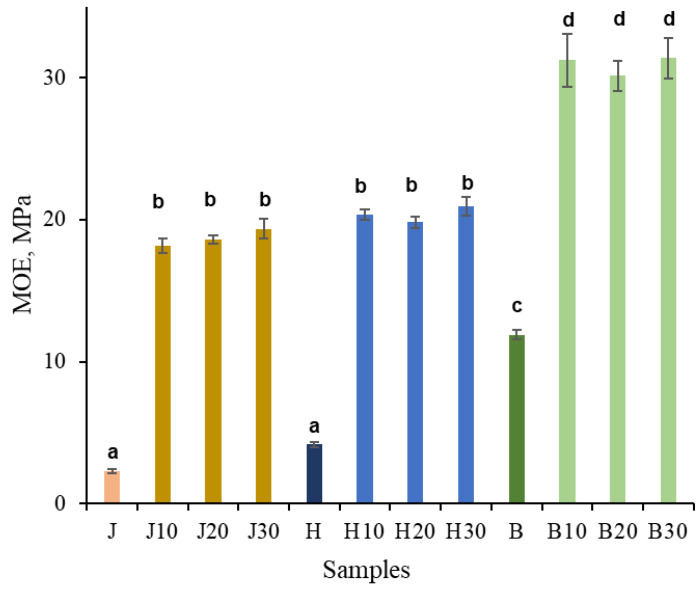
MOE (Young’s modulus) of untreated juniper (J, orange), hawthorn (H, blue), and birch (B, green), and at different times (10, 20, 30 min), NaOH-treated densified wood. Bars sharing the same letter showed no significant difference by ANOVA at *p* = 0.05 (*n* = 3).

As shown in Figure 4, according to the three-point bending results, untreated juniper, hawthorn, and birch exhibited moduli of elasticity of 2.3, 4.1, and 12 GPa, respectively. The MOE values of untreated juniper and hawthorn were not statistically significantly different. After NaOH pretreatment followed by densification, the modulus of elasticity increased approximately eightfold for juniper, fivefold for hawthorn, and 2.5-fold for birch. As a result, the MOE values were approximately 18 and 20 GPa for juniper and hawthorn, respectively, which were not statistically significantly different, and approximately 31 GPa for birch. The duration of NaOH pretreatment had no statistically significant effect on the MOE values.

For comparison, hydrothermally densified juniper wood reported in previous work reached a similar density increase but exhibited substantially lower stiffness (MOE ≈ 8 GPa), while Kraft-type pretreatment (NaOH + Na_2_S) resulted in MOE values of ~13 GPa at comparable density [13]. The higher MOE values obtained here (~18–19 GPa) at similar final density indicate that NaOH-only pretreatment contributes to mechanical enhancement beyond densification alone, likely through selective chemical activation of the cell-wall matrix.

**Figure 5:**
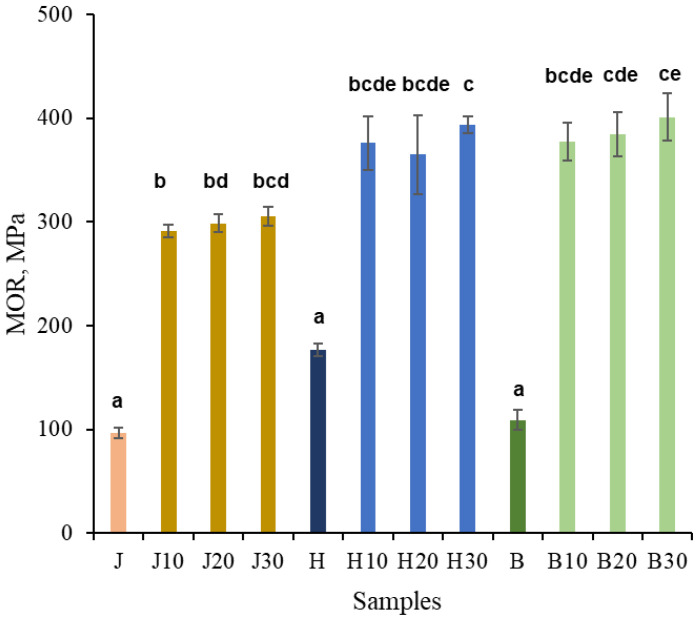
MOR (strength) of untreated juniper (J, orange), hawthorn (H, blue), and birch (B, green), and at different times (10, 20, 30 min), NaOH-treated densified wood. Bars sharing the same letter showed no significant difference by ANOVA at *p* = 0.05 (*n* = 3).

As seen in Figure 5, after NaOH pretreatment followed by densification, the MOR increased by approximately threefold for juniper and by about 2.5-fold for both hawthorn and birch, reaching values of approximately 300, 390, and 400 MPa, respectively. The duration of NaOH pretreatment had no statistically significant effect on MOR. The MOR values of densified hawthorn and birch did not differ significantly from each other, whereas juniper samples showed statistically significant differences compared to some hawthorn and birch samples. Minor variations can be attributed to anatomical variability; however, all treated samples converged toward a similar compacted state once sufficient plasticization was achieved.

In previous studies, hydrothermally densified juniper wood reached MOR values of approximately 150 MPa, while Kraft-type pretreatment yielded MOR values of ~180–190 MPa at similar densities [13]. In contrast, NaOH-only pretreatment in the present study enabled MOR values of up to ~300 MPa, despite comparable final density (~1200 kg m^−3^), demonstrating that chemical modification rather than densification alone governs the observed strength improvement.

## 4. Discussion

In the following sections, the effects of NaOH pretreatment on wood structure and properties are discussed in relation to densification mechanisms reported in the literature.

### 4.1. Microstructural Observations After NaOH Pretreatment and Densification

The SEM observations indicate that NaOH pretreatment followed by hot pressing results in a highly compact microstructure, characterized by near-complete collapse of lumens, vessels, and tracheids and close packing of the cell-wall material (Figure 1B,D,F). Such extensive microstructural consolidation is a key prerequisite for achieving high density and enhanced mechanical performance in densified wood, as also reported in previous studies on chemically and physically densified wood [2,3,14,35,49,58].

The observed structural changes can be attributed to alkaline-induced activation of the cell-wall matrix. Sodium hydroxide pretreatment promotes softening and increased mobility of lignin-rich regions and the middle lamella, which reduces resistance to cell-wall deformation during compression [11,59,60]. As a result, the integrity of the native cellular structure is weakened, facilitating lumen distortion, collapse, and intimate contact between adjacent cell walls during hot pressing. Upon drying under pressure, these newly formed contacts become stabilized, contributing to irreversible densification and reduced elastic recovery [12,49,61,62].

The microstructural features observed here are consistent with those reported for densified wood produced using other chemical or physicochemical pretreatment strategies, including sulfur-containing systems [11,13,14,35], deep eutectic solvents [15], acid or alkyl pretreatments [63], and hydrothermal or steam-based approaches [64]. In these studies, enhanced cell-wall deformability and lumen collapse are likewise identified as the dominant structural mechanisms enabling effective densification [62]. The present results demonstrate that comparable microstructural consolidation can be achieved using a simplified NaOH-only pretreatment, without the need for sulfite-based chemistry.

### 4.2. Chemical Activation of the Cell-Wall Matrix

FTIR and chemical analyses indicate that NaOH pretreatment primarily induces hemicellulose deacetylation and structural modification of lignin, rather than extensive removal of the main wood polymers. The disappearance or strong reduction in the absorption bands at ~1720 cm^−1^ and ~2959 cm^−1^ is consistent with the removal of acetyl side groups from hemicelluloses, particularly xylans. This interpretation is supported by HPLC results showing a pronounced decrease in acetic acid released after acid hydrolysis, while the overall hemicellulose content remains largely unchanged (Table 1) [12].

Removal of acetyl groups is known to increase polysaccharide chain mobility and reduce steric constraints within the cell-wall matrix, thereby facilitating deformation under compression [65]. In the present case, such deacetylation likely contributes to cell-wall softening and enables more effective densification, as reflected by the extensive lumen collapse observed by SEM.

The observed changes in extractive content further reflect differences in extractive composition among the studied wood species. Juniper contains a higher proportion of alkali-soluble extractives, such as resin acids and terpenoid compounds, which are readily removed during NaOH treatment [66,67]. In contrast, hawthorn extractives are dominated by more alkali-resistant phenolic compounds, including tannins and flavonoid-type structures, resulting in minimal extractive loss [68]. Birch exhibits intermediate behavior, consistent with partial removal of alkali-soluble components alongside more stable extractives [69].

Importantly, the relative increase in cellulose content observed after treatment is mainly a consequence of the reduction of extractives and deacetylated hemicellulose side groups, rather than cellulose enrichment through polymer removal [70].

FTIR results further indicate partial modification of lignin structure. The decrease in the ~1510 and ~1483 cm^−1^ bands observed mainly in juniper samples is attributed to alkaline-induced cleavage of β-O-4 linkages in guaiacyl lignin [55]. Importantly, Klason lignin analysis shows that the total lignin content remains essentially unchanged (Table 1), indicating structural modification rather than delignification.

Taken together, the combined FTIR, HPLC, Soxhlet, and Klason lignin results show that NaOH pretreatment selectively modifies side groups and interunit linkages while preserving the bulk polymer framework. This supports the view that effective densification does not require extensive polymer removal but can be achieved through selective chemical activation that enhances cell-wall mobility [3]. Importantly, recent reviews of wood densification and chemical pretreatment consistently conclude that extensive polymer removal is not a prerequisite for achieving high mechanical performance. Instead, two factors are identified as dominant: (i) effective collapse and consolidation of the cellular structure and (ii) chemical activation of the cell-wall matrix, particularly through hemicellulose deacetylation, which increases polymer mobility and reduces resistance to irreversible deformation [3]. Within this framework, changes in bulk polymer content are often of secondary importance, provided that sufficient cell-wall softening is achieved to enable intimate cell-wall contact during compression. The present results are fully consistent with this concept, as NaOH pretreatment primarily induces deacetylation and limited lignin modification while preserving the overall polymer framework.

In contrast, pretreatments employing sulfur-containing additives or anthraquinone typically lead to more severe chemical changes, including substantial hemicellulose loss and partial lignin removal [11,13,14,49]. The present results demonstrate that a NaOH-only approach can promote cell-wall plasticization and densification without invoking such high-severity chemistry.

### 4.3. Relationship Between Microstructural Consolidation, Chemical Modification, and Mechanical Performance

The substantial increase in density and mechanical properties observed after NaOH-only pretreatment and densification can be directly linked to the combined microstructural and chemical changes identified by SEM and FTIR analyses. SEM observations demonstrate extensive lumen collapse and close packing of the cell-wall material, indicating effective microstructural consolidation of the wood matrix. Such consolidation is a necessary prerequisite for achieving high stiffness and strength, as load transfer in densified wood is increasingly governed by intercellular contact area and cell-wall continuity rather than by the native cellular architecture.

A comparison with previously reported juniper densification routes shows that density increase alone does not fully explain the observed mechanical performance. In earlier work, hydrothermally densified juniper reached ~1050 kg m^−3^ with MOR around 150 MPa and MOE ~8 GPa, while Kraft-type pretreatment (NaOH + Na_2_S) enabled higher densification (~1200 kg m^−3^) and improved properties (MOR ~180–190 MPa; MOE ~13 GPa). In contrast, the NaOH-only pretreatment applied here achieves a similar density (~1200 kg m^−3^) but results in substantially higher mechanical performance, with MOR up to ~300 MPa and MOE ~18–19 GPa [13,43].

These results indicate that NaOH-only pretreatment provides a mechanical advantage that cannot be attributed solely to densification level. Instead, selective hemicellulose deacetylation and lignin structural modification likely promote more effective cell-wall plasticization and intercellular bonding during hot pressing. Although comparable reference data are not yet available for birch and hawthorn, the similar chemical and microstructural responses observed across species suggest that this effect is not limited to juniper.

The achieved density levels are comparable to those obtained using more chemically complex pretreatment routes. Pretreatment of wood with NaOH yielded density values (1200–1300 kg m^−3^) comparable to those reported after sulfite-based pretreatments [11,13,14,35,43,61] and deep eutectic solvent treatments [15]. Importantly, the present density values fall within the upper range reported for chemically pretreated densified wood, despite the absence of sulfite-based delignification or multi-component chemical systems. In contrast, pretreatments based on steam [17,64], acid, or alkyl treatments [63] reported by other authors generally resulted in lower density (800–1000 kg m^−3^) increases in wood samples. These comparisons indicate that extensive delignification is not a prerequisite for achieving near-cell-wall-level densities.

FTIR and chemical analyses provide further insight into the mechanisms underlying this behavior. Although NaOH pretreatment caused only minor reductions in the hemicellulose and lignin contents (approximately 1–2%) (Table 1), it likely induced structural modifications of lignin rather than extensive degradation. In parallel, FTIR results indicate deacetylation of hemicelluloses, which reduces steric constraints and increases chain mobility within the cell-wall matrix. Together, these changes promote softening of lignin-rich regions and the middle lamella, facilitating irreversible cell-wall deformation during hot pressing. Alkaline treatment can promote lignin softening, partial depolymerization, and subsequent repolymerization, increasing its mobility and reactivity. During subsequent densification, this plasticized lignin may form new inter- and intramolecular interactions or covalent bonds, thereby enhancing inter-fiber bonding and load transfer. These combined effects are considered to play an important role in the substantial improvement of the mechanical properties of the densified wood [16,71].

In comparison, the MOE values obtained in the present study (up to ~30 GPa) are comparable to or exceed those reported for many sulfur-based and hydrothermal pretreatment routes, while avoiding the high severity required to achieve extreme stiffness values. In other studies, on poplar and spruce densification using various pretreatment methods (including sulfite-based), similar or lower Young’s modulus values of approximately 22–30 GPa have been reported [7,15,35,63,64,72]. Significantly higher MOE values have also been achieved for poplar pretreated with sodium hydroxide and sodium bisulfite, reaching up to 120 GPa, depending on the pretreatment and densification temperatures [73]. This indicates that NaOH-only pretreatment enables efficient stiffness enhancement under comparatively mild conditions.

A similar trend is observed for strength. In other studies, lower absolute strength values of approximately 150 MPa were reported for densified poplar wood subjected to hydrothermal densification [64] or NaOH pretreatment [63]. For poplar wood pretreated with sodium hydroxide in combination with sodium sulfite (Na_2_SO_3_), MOR values typically range between approximately 150 and 300 MPa, depending on pretreatment severity and densification conditions [63,72,73], while for sodium hydroxide and sodium bisulfite, the modulus of rupture has been reported to range between approximately 200 and 400 MPa, depending on the pretreatment conditions and densification temperature [73]. In contrast, sulfite cooking pretreatment followed by sodium silicate impregnation increased the strength of densified poplar to about 300 MPa [7]. For softwood species, densified spruce reached strength values of approximately 220 MPa [35]. In comparison, deep eutectic solvent pretreatment increased the strength of densified pine to around 180 MPa [15]. Notably, densified cedar treated using deep eutectic solvents has been reported to reach maximum MOR values of only about 125 MPa, highlighting the comparatively limited strengthening achievable with DES-based pretreatments for some softwood species [2].

Overall, the results indicate that NaOH-only pretreatment is sufficiently effective to plasticize lignin and promote the formation of new interactions during densification. The mechanical properties of all wood samples improved substantially even at the shortest NaOH pretreatment duration, suggesting that longer pretreatment times are not required. Compared with literature data, the present NaOH-only approach achieves density, stiffness, and strength levels comparable to or exceeding those reported for more chemically complex pretreatments, while relying on a simplified, sulfur-free system. The obtained physical and mechanical properties are comparable to those reported in the literature for chemically pretreated densified wood and demonstrate the potential of this approach for the development of high-performance wood-based materials.

## 5. Conclusions

This study demonstrates that NaOH-only pretreatment is an effective approach for enabling wood densification by sufficiently plasticizing lignin and facilitating cell wall deformation. The results show that effective densification can be achieved not through extensive polymer removal, but rather through selective chemical activation of the cell-wall matrix. As a result, all investigated wood species exhibited substantial improvements in density and mechanical properties, with comparable increases in modulus of elasticity and modulus of rupture already achieved at the shortest NaOH pretreatment duration, indicating that longer treatment times are unnecessary for both softwood and hardwood species. Chemical analyses indicate that NaOH pretreatment causes only minor structural changes, primarily associated with hemicellulose deacetylation and lignin modification, while the overall composition of the main wood polymers remains largely unchanged. This confirms that hemicellulose deacetylation and cell-wall softening, rather than delignification, are sufficient to enable irreversible densification and mechanical enhancement. Overall, these findings demonstrate that a simplified, NaOH-only, and sulfite-free pretreatment can deliver mechanical performance comparable to more complex chemical routes while relying on milder chemistry and preserving the bulk polymer framework of wood.

## Figures and Tables

**Table 1:** Chemical characterization of untreated juniper (J), hawthorn (H), and birch (B), and at different times (10, 20, 30 min), NaOH-treated densified wood.

Sample	J	J10	J20	J30	H	H10	H20	H30	B	B10	B20	B30
Component	Quantity, %											
Extractives	3.01	2.81	0.53	0.75	1.00	1.03	1.01	1.02	1.52	1.03	1.04	1.03
Glucose	38.85	42.61	44.75	44.92	40.77	45.93	47.39	46.90	37.56	40.68	41.40	42.32
Xylose	8.43	8.58	8.61	8.60	23.15	22.70	22.62	22.66	25.15	24.91	24.92	24.75
Ather hemicelluloses	12.7	11.87	11.69	11.71	5.61	3.42	2.50	2.51	7.52	7.11	7.11	6.92
Acid-soluble lignin	0.9	0.87	0.90	0.88	0.62	1.84	1.73	1.83	0.56	0.59	0.54	0.60
Acid-insoluble lignin	31.49	30.32	30.39	30.32	22.54	21.65	21.60	21.22	23.46	22.51	22.64	22.59
Inorganic part	1.96	1.98	1.97	1.98	1.53	1.18	1.13	1.20	1.23	1.02	1.04	0.66
Acetic acid	1.78	0.15	0.10	0.10	3.59	1.69	1.13	1.17	2.15	0.92	0.84	0.80
Others	1.36	1.20	1.28	1.50	2.15	1.29	1.30	1.74	1.23	1.51	1.22	1.22

Note: To improve readability, hemicellulose components (except xylan) are grouped and standard deviations are omitted in this table. The full compositional dataset with statistical details is available in the Supplementary Materials.

## Data Availability

The original contributions presented in this study are included in the article. Further inquiries can be directed to the corresponding author.

## Supplementary Materials

The following supporting information can be downloaded at: https://www.mdpi.com/article/10.3390/polym18030312/s1, Table S1. Chemical characterization of untreated juniper (J), hawthorn (H), and birch (B), and at different times (10, 20, 30 min), NaOH-treated densified wood.

## Abbreviations

The following abbreviations are used in this manuscript:

J	Untreated juniper wood
J10; J20; J30	NaOH treated juniper wood in different time (10; 20; 30 min)
H	Untreated hawthorn wood
H10; H20; H30	NaOH treated hawthorn wood in different time (10; 20; 30 min)
B	Untreated birch wood
B10; B20; B30	NaOH treated birch wood in different time (10; 20; 30 min)
MOE	Modulus of elasticity
MOR	Modulus of rapture
SEM	Scanning Electron Microscopy
FTIR	Fourier Transform Infrared Spectroscopy
SE	Standard error
