# Supplementary material for: NaOH-Only Pretreated Wood Densification: A Simplified Sulfite-Free Route Across Wood Species
Source: Polymers (Basel). 2026 Jan 23;18(3):312. doi: 10.3390/polym18030312 (PMC12899162; doi:10.3390/polym18030312)
Supplement: Supplementary file 1 [file polymers-18-00312-s001.zip › polymers-4087848-supplementary.pdf]

Table S1. Chemical characterization of untreated juniper (J), hawthorn (H), and birch (B), and at different times (10, 20, 30 min), NaOH-treated densified wood.

**Sample**	**J**		**J10**		**J20**		**J30**		**H**		**H10**		**H20**		**H30**		**B**		**B10**		**B20**		**B30**	
Component	Quantity		Quantity		Quantity		Quantity		Quantity		Quantity		Quantity		Quantity		Quantity		Quantity		Quantity		Quantity	
	% a.d.m.	±	% a.d.m.	±	% a.d.m.	±	% a.d.m.	±	% a.d.m.	±	% a.d.m.	±	% a.d.m.	±	% a.d.m.	±	% a.d.m.	±	% a.d.m.	±	% a.d.m.	±	% a.d.m.	±
**Extractives**	**3.01**	*0.02*	**2.81**	*0.01*	**0.53**	*0.02*	**0.75**	*0.02*	**1.00**	*0.03*	**1.03**	*0.01*	**1.01**	*0.01*	**1.02**	*0.02*	**1.52**	*0.01*	**1.03**	*0.01*	**1.04**	*0.02*	**1.03**	*0.01*
**Glucose**	**38.85**	*0.48*	**42.61**	*0.07*	**44.75**	*0.39*	**44.92**	*0.25*	**40.77**	*0.05*	**45.93**	*0.19*	**47.39**	*0.30*	**46.90**	*0.50*	**37.56**	*0.52*	**40.68**	*0.29*	**41.40**	*0.92*	**42.32**	*0.11*
**Xylose**	**8.43**	*0.14*	**8.58**	*0.01*	**8.61**	*0.15*	**8.60**	*0.14*	**23.15**	*0.03*	**22.70**	*0.19*	**22.62**	*0.28*	**22.66**	*0.05*	**25.15**	*0.15*	**24.91**	*0.13*	**24.92**	*0.79*	**24.75**	*0.07*
**Galactose**	**2.73**	*0.27*	**2.68**	*0.19*	**2.67**	*0.13*	**2.71**	*0.09*	**1.87**	*0.03*	**1.10**	*0.08*	**1.03**	*0.09*	**1.02**	*0.04*	**2.57**	*0.17*	**2.34**	*0.12*	**2.51**	*0.15*	**2.43**	*0.03*
**Arabinose**	**2.35**	*0.83*	**1.65**	*0.02*	**1.60**	*0.05*	**1.60**	*0.09*	**0.97**	*0.02*	**0.11**	*0.02*	**0.13**	*0.12*	**0.09**	*0.05*	**1.56**	*0.07*	**1.55**	*0.09*	**1.56**	*0.08*	**1.59**	*0.03*
**Mannose**	**7.63**	*0.61*	**7.55**	*0.24*	**7.42**	*0.20*	**7.39**	*0.08*	**2.78**	*0.08*	**2.21**	*0.01*	**1.35**	*0.08*	**1.40**	*0.17*	**3.39**	*0.12*	**3.22**	*0.19*	**3.04**	*0.07*	**2.89**	*0.17*
**Acid-soluble lignin**	**0.9**	*0.01*	**0.87**	*0.01*	**0.90**	*0.02*	**0.88**	*0.01*	**0.62**	*0.01*	**1.84**	*0.03*	**1.73**	*0.00*	**1.83**	*0.03*	**0.56**	*0.02*	**0.59**	*0.02*	**0.54**	*0.01*	**0.60**	*0.01*
**Acid-insoluble lignin**	**31.49**	*0.53*	**30.32**	*0.20*	**30.39**	*0.02*	**30.32**	*0.01*	**22.54**	*0.01*	**21.65**	*0.04*	**21.60**	*0.17*	**21.22**	*0.21*	**23.46**	*0.18*	**22.51**	*0.17*	**22.64**	*0.04*	**22.59**	*0.11*
**Neorganic part**	**1.96**	*0.01*	**1.98**	*0.01*	**1.97**	*0.01*	**1.98**	*0.03*	**1.53**	*0.09*	**1.18**	*0.01*	**1.13**	*0.02*	**1.20**	*0.01*	**1.23**	*0.04*	**1.02**	*0.04*	**1.04**	*0.06*	**0.66**	*0.05*
**Acitic acid**	**1.78**	*0.02*	**0.15**	*0.01*	**0.10**	*0.02*	**0.10**	*0.02*	**3.59**	*0.01*	**1.69**	*0.46*	**1.13**	*0.01*	**1.17**	*0.04*	**2.15**	*0.23*	**0.92**	*0.01*	**0.84**	*0.02*	**0.80**	*0.07*
**Others**	**1.36**	*0.02*	**1.20**	*0.01*	**1.28**	*0.03*	**1.50**	*0.02*	**2.15**	*0.01*	**1.29**	*0.01*	**1.30**	*0.03*	**1.74**	*0.02*	**1.23**	*0.02*	**1.51**	*0.01*	**1.22**	*0.03*	**1.22**	*0.02*
